# Donation after brain circulation determination of death

**DOI:** 10.1186/s12910-017-0173-1

**Published:** 2017-02-23

**Authors:** Anne L. Dalle Ave, James L. Bernat

**Affiliations:** 1 Ethics Unit, University hospital of Lausanne, Rue du Bugnon 21, 1011 Lausanne, Switzerland; 2 Neurology Department, Dartmouth-Hitchcock Medical Center, Lebanon, NH 03756 USA

**Keywords:** Transplantation, Ethics, Donation after circulatory determination of death [DCDD], Extracorporeal membrane oxygenation [ECMO], Brain criterion of death, Determination of death

## Abstract

**Background:**

The fundamental determinant of death in donation after circulatory determination of death is the cessation of brain circulation and function. We therefore propose the term *donation after brain circulation determination of death* [DBCDD].

**Results:**

In DBCDD, death is determined when the cessation of circulatory function is permanent but before it is irreversible, consistent with medical standards of death determination outside the context of organ donation. Safeguards to prevent error include that: 1] the possibility of auto-resuscitation has elapsed; 2] no brain circulation may resume after the determination of death; 3] complete circulatory cessation is verified; and 4] the cessation of brain function is permanent and complete. Death should be determined by the confirmation of the cessation of systemic circulation; the use of brain death tests is invalid and unnecessary.

Because this concept differs from current standards, consensus should be sought among stakeholders. The patient or surrogate should provide informed consent for organ donation by understanding the basis of the declaration of death.

**Conclusion:**

In cases of circulatory cessation, such as occurs in DBCDD, death can be defined as the permanent cessation of brain functions, determined by the permanent cessation of brain circulation.

## Background

Human death has been conceptualized traditionally as a unitary phenomenon characterized by the irreversible cessation of the three vital functions: respiration, circulation and brain function [1]. Advances in life-sustaining therapies have disrupted the unitary nature of death, because now, new technologies can support or restore some vital functions while others remain absent [2]. The development of mechanical ventilation in the 1950s permitted the maintenance of respiratory and, thereby, circulatory functions, despite the irreversible cessation of brain function [3]. That the three vital functions no longer are interdependent stimulated the development of the brain criterion of death [4].

Advances in resuscitation techniques, particularly cardiopulmonary resuscitation (CPR) and extracorporeal membrane oxygenation (ECMO)-assisted CPR [5–7] now allow recovery of patients formerly considered dead. In donation after circulatory determination of death (DCDD) programs, because of the need to optimize organ suitability for donation, death is declared shortly after circulatory cessation [8], a time which has provoked questions over the validity of death determination [9–13].

Novel resuscitation techniques and DCDD have shown the need for a more explicit analysis of death by raising several controversial questions. Is the DCDD patient actually dead at the moment death is pronounced given that the cessation of circulation may not be irreversible at that time? Is reliance on the permanent cessation of circulation sufficient grounds to declare death? [14] How are the phenomena of circulatory cessation and brain cessation related as criteria of death? Which criterion should be used to determine death in the context of DCDD programs: the circulatory criterion, as practiced in the U.S. [15], the brain criterion [16] as practiced in Switzerland [17], the Netherlands [18], and other European countries, or both? And how in ideal practice should death be determined?

We address these questions in this article and conclude that the permanent cessation of brain circulation is the critical element underlying both the circulatory and brain determination of death. We therefore propose a new term of this concept: organ *donation after brain circulation determination of death* (DBCDD).

## The fundamental criterion of death is the cessation of brain function

While the three vital functions (respiration, circulation and brain function) are interrelated and interdependent in the absence of medical intervention, the fundamental determinant of death is the irreversible cessation of brain function [19–21]. This state is achieved commonly by one of two pathways: (1) the permanent cessation of systemic (including brain) circulation after circulatory arrest; and (2) the cessation of brain circulation resulting from markedly increased intracranial pressure following a catastrophic brain injury [22].

Through the pathway of catastrophic brain injury, death is determined by the brain criterion of death, i.e. by the irreversible cessation of all clinical brain functions, including the brainstem [4, 23], while respiratory and circulatory functions remain sustained by mechanical ventilation. Through the pathway of circulatory cessation, the fundamental criterion of death is also the brain criterion. As a consequence of the cessation of systemic circulation, brain circulation ceases followed rapidly by a progressive neuronal destruction due to hypoxemia and ischemia [2], leading to the irreversible cessation of all brain function.

We offer two examples showing that, through the pathway of circulatory cessation, the bodily circulation most relevant to death determination is that to the brain. In the first example, a patient with cessation of systemic circulation has her brain circulation sustained by local ECMO. Her brain function remains intact because of the ongoing brain circulation, while her bodily circulation has otherwise ceased. The usual circulatory criterion of death, i.e. the cessation of systemic circulation, would declare this patient dead, despite the fact that she might be fully conscious.

In the second example, a DCDD donor has her systemic circulation sustained by ECMO, while brain circulation is blocked by either an inflated aortic occlusion balloon [24] or an aortic occlusive clamp [25]. Depending on the degree to which the balloon or the clamp excludes brain perfusion, the DCDD donor may be either dead or alive [26, 27]. These two examples show that, in the pathway of circulatory cessation, the relevant circulation in the determination of death is that to the brain.

## From irreversibility to permanency in the determination of death

Philosophical and biological concepts of death require irreversibility. Irreversibility means that, once circulation and respiration have ceased, they cannot restart spontaneously and *cannot* be restored by any available technologies [28]. Yet, the medical determination of death using the circulatory/respiratory criterion relies on the permanent cessation of respiration and circulation. Permanent means that the ceased functions *will not* recover because they will not restart spontaneously and no medical attempts will be made to restart them. Therefore, a noncongruence exists between the concept of death that requires an irreversible cessation of vital functions and the medical standard of death determination that requires only their permanent cessation [28].

In the context of donation after brain determination of death (DBDD), brain death is declared only once physicians have established the irreversible cessation of brain function [23]. Irreversibility depends on the fact that, despite advances in brain resuscitation [29], no available technology can restore brain function once death has been confirmed by brain death tests [23]. Brain death has traditionally been formulated as a retrospective determination that shows that all clinical brain functions have ceased.

In the context of circulatory cessation (including DBCDD), irreversibility is, at least to some extent, contingent on physician action or inaction [30]. Apart from scenarios in which irreversibility is obvious clinically (eg, decapitation, incineration, decomposition, and rigor mortis), physicians declare death before a state of irreversibility has been achieved. It is common, particularly in the intensive care setting, to determine death only a few minutes after circulation ceases [31].

The exact duration of complete circulatory cessation necessary to achieve the irreversible cessation of brain function remains unknown [22]. Outcome studies after cardiac arrest reveal that some brain functions can be restored by resuscitation maneuvers after as long as 20 [32] to 30 min [33] of no-flow, i.e. from the time from collapse to the initiation of CPR [16]. Animal studies show that some brain functions may be restored after as long as 60 min of circulatory cessation [34–36].

These data suggest that, in DBCDD programs, the no-touch period - i.e. the time from circulatory cessation to the declaration of death which is usually 2–20 min [19, 37–39] - is too short to have achieved an irreversible cessation of neuronal function necessary to determine brain death. Furthermore, new technologies such as ECMO are able to restore some brain functions after approximately 30 min of circulatory cessation. Thus, in the context of circulatory cessation (including DBCDD), death is often determined during a state of permanent cessation but before a state of irreversible cessation [14].

Apart from the DBCDD context, physicians’ usual determination of death using permanent cessation is justified when several conditions are fulfilled: 1) The possibility of auto-resuscitation has elapsed; 2) No action will be initiated to restore circulation after the determination of death; 3) Further elapsed time will permit confirming the certainty of death; 4) There are social reasons for physicians to declare death sooner rather than later; and 5) Brain function has permanently and completely ceased. Let us now analyze whether these justifications in non-donation settings are sufficient grounds to declare death before irreversibility in the DBCDD context.

1. The possibility of auto-resuscitation has elapsed
  Auto-resuscitation refers to the spontaneous return of heartbeat and circulation after asystole [2]. To avoid the possibility of a patient’s revival after having been declared dead, death cannot be declared until the possibility of auto-resuscitation has elapsed.
  The minimal no-touch period to wait to ensure the impossibility of auto-resuscitation is unknown [40]. In one comprehensive retrospective study of withdrawal of life-sustaining therapy, the investigators found not a single case of auto-resuscitation to restored circulation [41]. An observational study showed that ‘the longest period of cessation of arterial blood pressure before resumption was 89 sec [40]. While cardiac electrical activity persisted in some patients for more than 30 min after cardiac arrest, the authors concurred that ‘true autoresuscitation required the spontaneous return of circulation’ and not merely the return of cardiac electrical activity [2, 39–41]. By contrast, after failed CPR in the setting of unexpected cardiac arrest, auto-resuscitation to circulation remains possible after 7 min of cardiac arrest [41].
  Available data suggest that in controlled DCDD following withdrawal of life-sustaining therapy, a no-touch period of 5 min is sufficient to ensure the impossibility of auto-resuscitation. But in uncontrolled DCDD after failed resuscitative efforts in an unexpected cardiac arrest, a no-touch period for as long as 10 min may be necessary [42].
2. No action will be initiated to restore circulation after the determination of death
  Because after circulatory cessation, death usually is declared before the cessation of brain functions become irreversible, brain functions potentially could respond to the restoration of brain circulation [43]. To avoid this risk, the use of any procedures or technologies that can restore brain circulation should be avoided following the determination of death [19, 26, 43, 44]. DBCDD programs should avoid the use of post-mortem ECMO, as currently practiced in some controlled DBCDD programs [24, 25, 45–47] and several uncontrolled DBCDD programs [48, 49]. The post-mortem use of ECMO may retroactively invalidate the preceding death declaration by negating the necessary condition of permanent cessation of circulation.
  Similarly, the use of CPR after the declaration of death, as practiced in some uncontrolled DBCDD programs [48, 49], should be avoided, because it may restore brain circulation. Inflation of the lungs after the declaration of death, which some transplantation programs use when the lungs are planned to be procured, should be avoided, because it may stimulate the return of spontaneous heartbeat and circulation. Our current resuscitative technology has complicated the concept of irreversibility which now may be contingent on physician action or inaction [28].
**The case of cold organ preservation solution ECMO**
  The University of Pittsburgh Medical Center is developing an innovative uncontrolled DCDD protocol using post-mortem ECMO with cold organ preservation solution instead of blood to preserve organs [50]. In their protocol, brain perfusion is resumed after the declaration of death not with blood but with cold fluid. Although the cold solution cannot oxygenate the brain, the accompanying hypothermia slows brain neuronal metabolism. Hypothermia could invalidate the preceding determination of death by preventing the brain neuronal hypoxic-ischemic damage that allows death to be determined on the grounds of the permanent cessation of circulation. We therefore recommend avoiding the use of cold organ preservation solution ECMO.
3. Further elapsed time will permit confirming the certainty of death
  One controversy in declaring death prior to a state of irreversibility in DBCDD is that, because organ procurement rapidly follows the determination of death, the ability to confirm the certainty of death is diminished [10]. Because death in DBCDD is followed by organ procurement, the diagnosis of death must be certain [13].
  In DBDD, irreversibility is necessary to diagnose death, because of the ongoing circulation and the risk of confounded and reversible conditions that may mimic brain death [23]. In DBCDD, irreversibility is unnecessary to the diagnosis of death because the permanent cessation of circulation will always lead to the irreversible cessation of brain function. The risk of error in the determination of death in the context of circulatory cessation results only from whether circulatory cessation is total and whether the possibility of auto-resuscitation has elapsed.
  Because in the DBCDD context, the cessation of circulation must be total, a technique such as intra-arterial monitoring, Doppler flow study, or echocardiography showing the non-opening of the aortic valve should be required to confidently distinguish the complete absence of circulation from the presence of small degree of circulation [20].
4. There are social reasons for physicians to declare death sooner than later
  In cases of circulatory cessation, physicians have declared death prior to the moment of irreversibility for social reasons without creating controversy. Delaying the declaration of death for a few hours while awaiting clinical signs of irreversibility (lividity, rigidity), imposes unjustifiable harms on family members. Interference with the bereavement process and preparation of the body caused by the delay in the determination of death may produce emotional and psychological suffering.
  It was not until DBCDD standards of death determination had to be made explicit that any controversy arose. We believe that the social reasons to declare death prior to irreversibility also apply in the DBCDD context. Because the declaration of death is more consequential in the DBCDD context, there should be safeguards to assure that: 1) the possibility of auto-resuscitation has elapsed; 2) circulatory cessation is total; 3) no actions will be initiated after the declaration of death that may possibly resume brain circulation and/or function; and 4) brain functions must have ceased permanently and completely.
5. Brain functions have permanently and completely ceased
  One worry raised by critics of relying on permanent cessation to declare death in DBCDD is that the donor may suffer because organ procurement will happen before the state of brain function irreversibility [51]. These critics claim that whereas, a “premature” declaration of death may be inconsequential outside the DBCDD context, it is very consequential in DBCDD, because bodily integrity may be damaged by organ procurement. To avoid any risks of inducing suffering on DBCDD donors by surgical procedures [52], we must be certain that the declaration of death corresponds to the cessation of all brain functions, thereby excluding the possibility of feeling pain and awareness.
  The precise time interval after brain circulatory cessation necessary to ensure the complete cessation of brain function is unknown. There is a consensus among neuroscientists and neurologists that brain function is a necessary condition of awareness, and that normothermic patients who have completely lost systemic (and therefore brain) circulation for more than a few minutes have no brain function or awareness. Brain circulatory cessation for 5 min at normothermia thus ensures the permanent and complete cessation of all brain functions. This condition, in turn, ensures that the DBCDD donor cannot experience any pain, suffering or awareness.
  The clinical assessment of the absence of brain function in the context of circulatory cessation cannot be accomplished easily. Surface electroencephalogram (EEG) examination may show the absence of thalamocortical electrical activity, but cannot exclude residual activity within brain stem structures [10, 19, 51]. In a case series of bispectral (BIS) measurements during DBCDD, the BIS value fell quasi-instantly to zero after cardiac arrest [53]. In the observational study done by Dhanani et al., EEG activity stopped rapidly after circulatory cessation but persisted for 26 min in one patient. The authors concluded that the measured EEG activity in that case was artifact [40]. A recent literature review revealed that most analyzed studies showed the absence of consciousness and EEG activity within 30 s of circulatory cessation [54].
  The confirmation of the precise period of time necessary to achieve the permanent and complete cessation of brain function after circulatory cessation must be further studied. In the meantime, a no-touch period of 5 min seems sufficient to achieve the permanent and complete cessation of all brain functions.

## How is death determined in DBCDD?

In the context of circulatory cessation, death is determined by the confirmation of the cessation of circulation and respiration. In the context of DBCDD, the complete cessation of systemic circulation should be confirmed with certainty by the use of an arterial line, a Doppler, or an echocardiogram.

If any technologies or procedures are used that possibly could resume brain circulation after death declaration, it must be proven that brain circulation has been completely excluded [26]. However, there may be technical difficulties in interpreting brain blood flow studies to prove the complete absence of brain circulation, particularly in distinguishing zero blood flow from a very small amount of blood flow.

The use of accepted and validated brain death test batteries in the context of DBCDD is inapplicable and unnecessary [16]. In DBDD, brain death tests are valid only if reversible causes of brain function cessation have been excluded, which is unfeasible in DBCDD, as we have discussed elsewhere [16].

Furthermore, because systemic circulatory cessation will automatically cause brain circulation to cease, which will lead to the progressive destruction of neuronal tissue, it is unnecessary to use brain death tests in the context of DBCDD. The confirmation of the permanent absence of systemic circulation is thus sufficient to determine death. To ensure that the possibility of auto-resuscitation has elapsed, death should be declared only after at least 5 min of no-touch period in controlled DBCDD and 10 min in uncontrolled DBCDD.

## Conclusion

When systemic circulation ceases, the criterion of death is the permanent cessation of brain circulation. When organ donation is conducted, we call it *donation after brain circulation determination of death* or DBCDD. The brain circulation criterion of death becomes valid once six conditions are fulfilled as listed in Table 1.

**Table 1:** The conditions of validity of the brain criterion of death in the context of DBCDD

	Conditions of validity
1]	The possibility of autoresuscitation has elapsed:
	− no-touch period of 5 min in controlled DBCDD − no-touch period of 10 min in uncontrolled DBCDD
2]	No interventions that may resume brain circulation after the determination of death will be performed:
	− no CPR - no ECMO, unless the brain circulation is proven to have been securely excluded − no lung inflation
3]	The diagnostic of cessation of brain circulation is certain using at least one test:
	flat arterial line − no arterial Doppler signal − no opening of the aortic valve on echocardiography
4]	There is a permanent and complete cessation of all brain functions:
	− a minimal no-touch period of 5 min is required
5]	There is consensus that the determination of death may be based on the permanent cessation of brain circulation and function
6]	Informed consent for DBCDD has been obtained

Our proposal departs from medical standards of brain death in which the retrospective irreversible cessation of brain function has been a longstanding requirement. This prospective determination of permanent cessation of brain circulation and function is sufficient to declare that the DBCDD donor is dead. Our proposal has the advantage of unifying the phenomenon of death under the single brain criterion of death for both common pathways: circulatory cessation and primary brain damage.

Death could thus be defined as the permanent cessation of brain functions, determined either by the permanent cessation of brain circulation in cases of circulatory cessation (including to the brain) or by the irreversible cessation of brain functions in cases of severe brain injury.

## Abbreviations

CPR: Cardio-pulmonary resuscitation
DBCDD: Donation after brain circulation determination of death
DBDD: Donation after brain determination of death
DCDD: Donation after circulatory determination of death
ECMO: Extracorporeal membrane oxygenation
